# Holter-Derived Mean Heart Rate as a Predictor of Short-Term Clinical Risk in Patients Hospitalized With Acute Heart Failure

**DOI:** 10.14740/cr2189

**Published:** 2026-06-05

**Authors:** Vu Manh Tan, Pham Xuan Hung, Tran Vuong The Vinh, Nguyen Thi Van Khanh, Vo Thai Duy

**Affiliations:** a Department of Internal Medicine, Faculty of Medicine, Hai Phong University of Medicine and Pharmacy, Hai Phong, Vietnam; b Department of Anesthesiology, Hai Phong University of Medicine and Pharmacy, Hai Phong, Vietnam; c Department of Surgery, Hai Phong University of Medicine and Pharmacy, Hai Phong, Vietnam; d Can Tho University of Medicine and Pharmacy, Can Tho, Vietnam; e Department of Arrhythmology, Cho Ray Hospital, Ho Chi Minh City, Vietnam

**Keywords:** Acute heart failure, Holter monitoring, Heart rate, In-hospital mortality

## Abstract

**Background:**

Heart rate (HR) is a key marker of autonomic and hemodynamic stress in acute heart failure (AHF). Whether 24-h Holter-derived mean HR provides incremental prognostic value for short-term clinical outcomes during hospitalization remains unclear. This study evaluated the association between Holter-derived mean HR and short-term clinical outcomes in patients hospitalized with AHF.

**Methods:**

The study enrolled consecutive adults hospitalized with AHF at a tertiary hospital between October 2020 and June 2021. All participants underwent 24-h Holter monitoring during early hospitalization. Mean HR was categorized as < 70, 70–100, or > 100 beats per minute (bpm). Outcomes included in-hospital mortality, and all-cause mortality and readmission at 30, 60, and 90 days post-discharge. Two cardiologists blinded to outcomes interpreted Holter data. Statistical analyses were conducted to evaluate associations between mean HR categories and short-term clinical outcomes.

**Results:**

Ninety-four patients were included (mean age 66 ± 16.2 years; 56.4% female). In-hospital mortality was strongly associated with mean HR (4.8% for < 70 bpm vs. 31.3% for > 100 bpm; P = 0.023). A HR threshold > 100 bpm significantly increased in-hospital mortality (P = 0.012). In contrast, admission HR alone did not predict mortality (P = 0.573). Post-discharge mortality (1.1–4.3%) and readmission rates (5.3–13.8%) did not differ across HR categories.

**Conclusions:**

Holter-derived mean HR was a robust predictor of in-hospital mortality in patients hospitalized with AHF, outperforming single-time HR measurements. These findings may have potential relevance to perioperative risk assessment in patients with AHF, although prospective studies including surgical cohorts are required.

## Introduction

Acute heart failure (AHF) is a major cause of hospitalization and carries substantial morbidity and mortality. Beyond cardiology, AHF frequently intersects with perioperative and surgical care, as many patients require diagnostic procedures, urgent interventions, or elective operations during or shortly after their index admission. In these settings, hemodynamic instability and autonomic activation strongly influence risk. Heart rate (HR), a simple but fundamental physiological parameter, reflects sympathetic drive, myocardial oxygen demand, and circulatory stress, factors that are critically relevant for both acute management and perioperative risk assessment [1–3].

While routine clinical practice typically relies on single HR measurements recorded at admission or discharge, such isolated values may underestimate the true physiological burden in AHF. Tachycardia in this population fluctuates with congestion, neurohormonal activity, medication effects, and circadian variation. Continuous HR monitoring via 24-h Holter electrocardiography offers a more comprehensive assessment of autonomic and hemodynamic stress, providing an integrated measure of “heart rate burden.” Previous studies in chronic heart failure and non-cardiac surgery populations have shown that sustained tachycardia is associated with myocardial injury, arrhythmias, and early adverse outcomes. However, the prognostic value of mean HR specifically during the acute phase of AHF remains poorly defined, and its relevance to perioperative decision-making has not been adequately explored [4–12].

Understanding whether Holter-derived mean HR predicts short-term outcomes could have meaningful clinical implications. Identifying patients with unresolved sympathetic activation may help clinicians determine surgical timing, guide therapeutic optimization, and tailor perioperative monitoring strategies. Furthermore, incorporating continuous HR metrics into perioperative evaluation may complement traditional risk indices, which often overlook dynamic physiological instability.

The study focuses on patients hospitalized with AHF and does not specifically evaluate patients undergoing surgical or invasive procedures. Perioperative considerations are discussed to provide clinical context, as AHF frequently intersects with perioperative decision-making in routine practice. The study aimed to evaluate the association between 24-h Holter-derived mean HR and short-term clinical outcomes—including in-hospital mortality, post-discharge mortality, and readmission—in patients hospitalized with AHF, with specific attention to its potential role in perioperative risk stratification.

## Materials and Methods

### Study design and participants

The study was an observational study; written informed consent was obtained from all participants. The study was approved by the Institutional Review Board of Hai Phong University Hospital, Vietnam and was conducted in compliance with the ethical standards of the responsible institution on human subjects and in accordance with the Helsinki Declaration. Patient anonymity and data confidentiality were strictly maintained throughout the study. Consecutive patients hospitalized with AHF were enrolled from October 2020 to June 2021. All participants underwent continuous 24-h Holter electrocardiographic monitoring during the early phase of hospitalization. Clinical outcomes were recorded during index admission and followed for 30, 60, and 90 days after discharge. No patients underwent acute surgical or invasive operative procedures during the index hospitalization or follow-up period.

### Inclusion criteria

Patients were eligible if they met the following criteria: 1) age ≥ 18 years; 2) hospitalization with a clinical diagnosis of AHF based on the 2016 European Society of Cardiology (ESC) guidelines [3], characterized by new-onset symptoms or acute decompensation of chronic heart failure; and 3) provision of informed consent for Holter monitoring and follow-up.

### Exclusion criteria

Patients were excluded if they had congenital heart disease; end-stage renal disease or serum creatinine > 3.5 mg/dL; recent cardiac surgery within 6 months; permanent pacemaker implantation or advanced atrioventricular conduction disturbances; or inability to undergo continuous Holter monitoring.

Patients with conditions known to cause marked HR abnormalities independent of AHF—such as uncontrolled hyperthyroidism, severe anemia requiring urgent intervention, or acute volume depletion—were excluded when these conditions were considered the primary drivers of tachycardia or bradycardia.

Competitive or endurance athletes were not included, as the study population consisted exclusively of hospitalized patients with AHF. Patients with extreme sinus bradycardia (< 40 beats per minute (bpm)) or clinically significant bradyarrhythmias requiring pacing therapy were also excluded.

### Study procedures

All consecutive patients admitted with symptoms suggestive of AHF were screened. AHF diagnosis was established according to the 2016 ESC criteria. Eligible patients were enrolled within 24 h of admission after providing written informed consent, and reasons for exclusion were documented.

Twenty-four-hour ambulatory electrocardiogram (ECG) monitoring was performed using a three-channel digital Holter recorder (Philips Digitrak XT, Philips, Amsterdam, Netherlands), compliant with standard ambulatory ECG recording specifications. Recordings were analyzed using dedicated manufacturer software, between 48 and 96 h of hospitalization once patients were clinically stable. Participants continued routine ward activities while nursing staff ensured lead adherence. Holter recordings were analyzed with dedicated software. Two cardiologists blinded to clinical outcomes independently reviewed all tracings. Extracted variables included mean, minimum, and maximum HR; rhythm type; atrial and ventricular ectopy; and presence of nonsustained ventricular tachycardia or conduction abnormalities. The study classified patients into three mean HR categories (< 70 bpm, 70–100 bpm, and > 100 bpm) based on several main considerations. A target HR of < 70 bpm is recommended by the ESC for the management of heart failure, making it a clinically meaningful threshold. A HR > 100 bpm represents the conventional cutoff for defining sinus tachycardia and is also used as a treatment threshold in patients with atrial fibrillation [3, 13].

During hospitalization, patients received standard-of-care treatment based on national and ESC guidelines. Clinical data, including medication use, hemodynamic status, repeat vital signs (particularly discharge HR), and laboratory reassessments, were recorded. In-hospital mortality was documented prospectively.

Post-discharge follow-up was performed at 30, 60, and 90 days through review of medical records and telephone interviews with patients or family members, with verification from external facilities when necessary. Outcomes included vital status, date and cause of death, all-cause readmission, and emergency department visits. All outcome assessors were blinded to Holter-derived HR groups.

Data quality was ensured through double-checking of all case-report forms, independent Holter review, and active follow-up, resulting in > 95% completeness. HR cut points were predefined a priori based on established clinical guidelines and conventions and were not derived from *post hoc* data analysis.

### Outcomes

Outcomes included all-cause mortality at 30, 60, and 90 days after discharge and all-cause readmission within the same time intervals. Readmission was defined as any unplanned hospitalization for any cause. Mortality and readmission events were ascertained through review of electronic medical records and structured follow-up telephone interviews with patients or family members. For the purpose of examining HR-related risk, outcomes were analyzed according to 24-h mean HR categories (< 70 bpm, 70–100 bpm, and > 100 bpm) as well as dichotomized cutoffs (< 70 vs. ≥ 70 bpm; ≤ 100 vs. > 100 bpm). All outcome assessors were blinded to Holter-derived HR groupings.

### Statistical analysis

Data were expressed as mean ± standard deviation or median with interquartile range (IQR), depending on distribution. The Shapiro–Wilk test was used to assess normality of continuous variables. Group comparisons across the three predefined mean HR categories (< 70 bpm, 70–100 bpm, > 100 bpm) were conducted using one-way analysis of variance (ANOVA) or the Kruskal–Wallis test for continuous variables, and the Chi-square test or Fisher’s exact test for categorical variables, as appropriate. Comparisons between dichotomized HR thresholds (≤ 100 vs. > 100 bpm and < 70 vs. ≥ 70 bpm) followed the same approach. Differences between admission and discharge vital signs were assessed using paired *t*-tests or Wilcoxon signed-rank tests.

Associations between HR categories and clinical outcomes, including in-hospital mortality, post-discharge mortality at 30, 60, and 90 days, and readmission, were evaluated using Chi-square or Fisher’s exact tests. Because of the limited number of outcome events, no multivariable regression model was constructed. All statistical tests were two-sided, and a P value < 0.05 was considered statistically significant. Missing data were handled by complete-case analysis, and overall data completeness exceeded 95%. Because of the limited number of in-hospital mortality events, multivariable regression analysis was not performed to avoid model overfitting and unstable estimates.

## Results

### Patient characteristics

A total of 94 patients were included and stratified into three groups based on mean Holter-derived HR: < 70 bpm (n = 21), 70–100 bpm (n = 57), and > 100 bpm (n = 16). Sex distribution was similar across groups (P = 0.084), although males predominated in the > 100 bpm group (26.8%). Age profiles did not differ significantly (P = 0.163); patients aged 60–80 years represented the largest proportion in all groups.

Comorbidities were common, with hypertension (57.4%) and dyslipidemia (64.9%) being most prevalent. Dyslipidemia (P = 0.005) and hypertension (P = 0.023) showed significant variation across HR groups, both being most frequent among patients with mean HR 70–100 bpm. Dilated cardiomyopathy tended to be more common in those with HR > 100 bpm (40.0%), though not statistically significant (P = 0.055). The distribution of other comorbidities—including coronary artery disease, valvular heart disease, chronic kidney disease, and diabetes mellitus—did not differ significantly.

Most patients presented with advanced symptoms, with New York Heart Association (NYHA) class III–IV predominating across all groups (P = 0.095). Echocardiographic parameters were similar between groups. Median left ventricular ejection fraction (LVEF) ranged from 30% to 38% (P = 0.246), and the proportion with LVEF < 40% did not differ significantly. Beta-blocker use during hospitalization was similar across mean HR categories (P = 0.132).

Biomarkers including N-terminal pro–B-type natriuretic peptide (NT-proBNP, P = 0.150) and troponin-T (P = 0.828) showed no statistical differences among the HR groups, although both tended to be higher in patients with mean HR > 100 bpm. Overall, the three HR groups demonstrated broadly comparable baseline characteristics aside from a higher prevalence of hypertension and dyslipidemia in the intermediate HR group and a tendency toward more dilated cardiomyopathy in those with tachycardia (Table 1).

**Table 1:** Baseline Characteristics of the Study Population According to Mean 24-H HR Categories

Characteristic	< 70 bpm (n = 21)	70–100 bpm (n = 57)	> 100 bpm (n = 16)	P value
Gender				0.084
Male	8 (19.5%)	22 (53.7%)	11 (26.8%)	
Female	13 (24.5%)	35 (66.0%)	5 (9.4%)	
Age groups				0.163
< 60 years	3 (10.0%)	20 (66.7%)	7 (23.3%)	
60–80 years	11 (24.4%)	26 (57.8%)	8 (17.8%)	
> 80 years	7 (36.8%)	11 (57.9%)	1 (5.3%)	
Comorbidities				
Body mass index (kg/m^2^) > 25	6 (16.2%)	23 (62.2%)	8 (21.6%)	0.405
Hypertension	16 (29.6%)	33 (61.1%)	5 (9.3%)	0.023
Dyslipidemia	17 (27.9%)	39 (63.9%)	5 (8.2%)	0.005
Coronary artery disease	17 (26.6%)	38 (59.4%)	9 (14.1%)	0.261
Dilated cardiomyopathy	2 (13.3%)	7 (46.7%)	6 (40.0%)	0.055
Valvular heart disease	6 (16.7%)	26 (72.2%)	4 (11.1%)	0.189
Chronic kidney disease	4 (26.7%)	11 (73.3%)	0	0.148
Diabetes mellitus	3 (11.1%)	19 (70.4%)	5 (18.5%)	0.249
Clinical severity				
NYHA III–IV	16	54	14	0.095
Beta-blocker use	15 (71.4%)	35 (61.4%)	14 (87.5%)	0.132
Echocardiography and biomarkers				
LVEF (%), median (IQR)	33.6 (23–41)	38 (31–50)	30 (21.9–56.5)	0.246
LVEF < 40%	14 (14.9%)	31 (33.0%)	11 (11.7%)	0.596
NT-proBNP (pg/mL)	3,276 (1,294–10,190)	4,962 (2,929–16,440)	6,507 (4,219–8,683)	0.150
Troponin-T (ng/L)	35.5 (15.5–115)	38.5 (19.3–82.2)	34.9 (22–46.4)	0.828

Data were presented as median and interquartile range (IQR) or n (%). HR: heart rate; bpm: beats per minute; NYHA: New York Heart Association; LVEF: left ventricular ejection fraction; NT-proBNP: N-terminal pro–B-type natriuretic peptide.

### Readmission rates by HR categories

Across the three HR categories (< 70, 70–100, and > 100 bpm), readmission rates remained similar at each follow-up interval. Thirty-day readmission occurred in 13 patients (13.8%), with nearly identical proportions across HR groups (14–18%). Sixty-day readmission was uncommon (5.3%) and showed no meaningful difference between HR groups. At 90 days, readmission occurred in eight patients (8.5%), again without a statistically significant association with HR groups. Overall, these results indicated that HR, regardless of measurement method, was not associated with early or late readmission among patients hospitalized with AHF (Table 2).

**Table 2:** Readmission Rates by Heart Rates

Outcome	Total (n = 94)	< 70 bpm	70–100 bpm	> 100 bpm	P value
30-day readmission	13 (13.8%)	3 (14.3%)	8 (14.0%)	2 (18.2%)	> 0.05
60-day readmission	5 (5.3%)	1 (5.0%)	4 (8.3%)	0 (0%)	> 0.05
90-day readmission	8 (8.5%)	4 (19.0%)	4 (8.3%)	0 (0%)	> 0.05

bpm: beats per minute.

### In-hospital and post-discharge mortality by HR

In-hospital mortality showed a strong and statistically significant association with mean 24-h HR categories. Mortality increased sharply across the three HR groups, from 4.8% in patients with mean HR < 70 bpm to 31.3% in those with HR > 100 bpm (P = 0.023). When HR was dichotomized at 100 bpm, patients with HR > 100 bpm had a markedly higher in-hospital mortality rate compared with those ≤ 100 bpm (P = 0.012). In contrast, admission HR alone was not significantly associated with in-hospital mortality (P = 0.573), indicating that continuous HR monitoring (mean HR) was a more reliable predictor of early mortality than single-time-point measurements.

Post-discharge mortality was low across all follow-up intervals. Thirty-day mortality occurred in four patients (all within the 70–100 bpm group), while 60-day and 90-day mortality were 1.1% each. Importantly, there were no statistically significant differences in 30-day, 60-day, or 90-day mortality across HR groups (Table 3).

**Table 3:** In-Hospital and Post-Discharge Mortality by Heart Rate

Outcomes	Total	< 70 bpm	70–100 bpm	> 100 bpm	P value
In-hospital mortality^a^	10 (10.6%)	1 (4.8%)	4 (7.5%)	5 (31.3%)	0.023
Admission HR and in-hospital mortality	—	2	4	4	0.573
30-day mortality	4 (4.3%)	0	4	0	> 0.05
60-day mortality	1 (1.1%)	0	1	0	> 0.05
90-day mortality	1 (1.1%)	1	0	0	> 0.05

^a^When dichotomized at 100 bpm (≤ 100 vs > 100), in-hospital mortality occurred in five patients in each group; however, due to the difference in group sizes (≤ 100 bpm: n = 78; > 100 bpm: n = 16), the corresponding mortality rates were 6.4% and 31.3%, respectively (P = 0.012). HR: heart rate; bpm: beats per minute.

## Discussion

The study showed that 24-h Holter-derived mean HR was strongly associated with in-hospital mortality, whereas HR measured at a single time point, either at admission or at discharge, showed a weaker prognostic relationship. Patients with a mean HR greater than 100 bpm had the highest in-hospital mortality, supporting the concept that sustained tachycardia reflects unresolved hemodynamic stress and sympathetic activation during the acute decompensated phase. Once stabilized, however, the predictive value diminished, as mean HR did not correlate with 30-, 60-, or 90-day post-discharge mortality or with readmission. This pattern suggests that the prognostic influence of HR is time-dependent, exerting its greatest impact during periods of acute circulatory instability.

The association between mean HR and early mortality aligns with existing evidence linking tachycardia to increased myocardial oxygen demand, reduced diastolic filling time, impaired coronary perfusion, and enhanced arrhythmogenic potential. Beta-blocker therapy is an important modifier of HR in patients with AHF. In the present study, the proportion of patients receiving beta-blockers was similar across mean HR categories. Notably, patients in the highest mean HR group (> 100 bpm) had the highest beta-blocker use, yet also experienced the highest in-hospital mortality. These findings suggest that the association between elevated mean HR and adverse in-hospital outcomes is unlikely to be explained solely by differences in beta-blocker therapy and may reflect persistent physiological instability beyond rate-modifying treatment. In AHF, where preload, afterload, and neurohormonal activation are already severely perturbed, persistent elevation in HR exacerbates these maladaptive pathways, accelerating clinical deterioration. Continuous HR monitoring may therefore capture physiological stress that intermittent vital sign measurements fail to detect. Studies in both chronic heart failure and perioperative populations have similarly shown that sustained tachycardia predicts myocardial injury, arrhythmias, and early postoperative complications, highlighting HR’s role as a dynamic marker of cardiovascular stress rather than a static measure of autonomic tone [13–16].

These findings may have meaningful implications for perioperative and surgical risk assessment. Patients hospitalized with AHF frequently require diagnostic procedures, urgent surgical interventions, or time-sensitive operations where hemodynamic stability is essential. A persistently elevated mean HR during hospitalization may signal unresolved physiological stress that increases vulnerability to perioperative myocardial injury, arrhythmias, and postoperative decompensation [11]. Unlike a single preoperative HR measurement, 24-h mean HR incorporates circadian variation, nocturnal sympathetic surges, and fluctuations in volume status, thereby providing a more reliable indicator of surgical readiness [6]. Integrating mean HR into perioperative evaluation could help determine whether surgery should be postponed for further optimization or whether intensified intraoperative and postoperative monitoring is warranted [17].

Mean HR also has potential to enhance existing perioperative risk assessment tools, many of which do not adequately account for autonomic instability in heart failure [5]. Current indices such as the Revised Cardiac Risk Index rely heavily on comorbidities but may overlook dynamic physiological markers of instability. Holter-derived HR provides a simple, noninvasive, and widely available measure that could complement clinical judgment and guideline-directed strategies. For high-risk patients, HR-guided optimization, such as careful titration of beta-blockers, improved volume management, or correction of reversible triggers, may reduce perioperative complications and improve overall outcomes.

### Limitations

The study had some limitations. It was conducted at a single center with a modest sample size, which may limit generalizability and the ability to detect associations with low-frequency outcomes such as 60- or 90-day mortality. Holter monitoring was applied after initial stabilization, potentially underestimating extreme HR variability during early decompensation. Residual confounding is possible despite prospective data collection and blinded outcome assessment. Although beta-blocker use was descriptively similar across HR categories, the limited number of outcome events precluded robust stratified or multivariable analyses to formally assess interaction effects between pharmacotherapy and HR. Therefore, residual confounding related to medication dose, adherence, or treatment intensity cannot be completely excluded. Larger, multicenter studies are needed to validate these findings and evaluate whether integrating mean HR into risk prediction models improves prognostic accuracy in AHF.

### Conclusions

Holter-derived mean HR was a strong predictor of in-hospital mortality in patients hospitalized with AHF. Persistent tachycardia may identify patients with ongoing physiological instability. These findings may have potential implications for perioperative risk assessment; however, prospective studies including surgical cohorts are required.

## Data Availability

Any inquiries regarding supporting data availability of this study should be directed to the corresponding author.
